# Temporal small RNA transcriptome profiling unraveled partitioned miRNA expression in developing maize endosperms between reciprocal crosses

**DOI:** 10.3389/fpls.2015.00744

**Published:** 2015-09-15

**Authors:** Mingming Xin, Guanghui Yang, Yingyin Yao, Huiru Peng, Zhaorong Hu, Qixin Sun, Xiangfeng Wang, Zhongfu Ni

**Affiliations:** State Key Laboratory for Agrobiotechnology, Key Laboratory of Crop Heterosis Utilization (MOE), Beijing Key Laboratory of Crop Genetic Improvement, China Agricultural UniversityBeijing, China

**Keywords:** maize endosperm, miRNA profiling, partitioned expression, reciprocal cross, tandem miRNA

## Abstract

In angiosperms, the endosperm nurtures the embryo and provides nutrients for seed germination. To identify the expression pattern of small interfering RNA in the developing maize endosperm, we have performed high-throughput small RNA transcriptome sequencing of kernels at 0, 3, and 5 days after pollination (DAP) and endosperms at 7, 10, and 15 DAP using B73 and Mo17 reciprocal crosses in previous study. Here, we further explored these small RNA-seq data to investigate the potential roles of miRNAs in regulating the gene expression process. In total, 57 conserved miRNAs and 18 novel miRNAs were observed highly expressed in maize endosperm. Temporal expression profiling indicated that these miRNAs exhibited dynamic and partitioned expression patterns at different developmental stages between maize reciprocal crosses, and quantitative RT-PCR results further confirmed our observation. In addition, we found a subset of distinct tandem miRNAs are generated from a single stem-loop structure in maize that might be conserved in monocots. Furthermore, a SNP variation of *Zma*-miR408-5p at 11th base position was characterized between B73 and Mo17 which might lead to completely different functions in repressing targets. More interestingly, *Zma*-miR408-5p exhibited B73-biased expression pattern in the B73 and Mo17 reciprocal hybrid endosperms at 7, 10, and 15 DAP according to the reads abundance with SNPs and CAPS experiment. Together, this study suggests that miRNA plays a crucial role in regulating endosperm development, and exhibited distinct expression patterns in developing endosperm between maize reciprocal crosses.

## Introduction

In flowering plants, the triploid endosperm is derived from the fertilization of the central cell (2n) and the sperm cell (1n) (Yadegari and Drews, 2004). Although it is a terminal tissue, it plays a crucial role not in only accumulating nutrients but also in supporting the development of the diploid embryo. In maize, the endosperm initiates a phase of syncytial development upon fertilization, where the nucleus undergoes mitosis in the absence of cytokinesis. This process is followed by a period of cellularization at approximately 3 days after pollination (DAP), during which existing endosperm nuclei are bound by cell walls (Berger, 2003; Olsen, 2004; Sabelli and Larkins, 2009). Subsequently, intensive cell division occurs. Following these proliferative processes, the differentiation, and specialization of endosperm cells begins at ~4–6 DAP and results in four primary endosperm compartments: the starchy endosperm, the basal endosperm transfer layer, the aleurone, and the embryo-surrounding region. At approximately 10 DAP, nutrient uptake and zein storage protein synthesis initiate, coincident with the rapid increase of auxin accumulation. Finally at ~15 DAP, the rate of fresh weight grain reaches a peak (Lur and Setter, 1993).

Temporal transcriptome profiling has revealed that gene expression patterns are dramatically changed during the development of maize endosperms (Li et al., 2014). These development-dependent genes involved in distinct functional categories, including amino acid biosynthesis, carbohydrate biosynthesis, and energy reserve metabolic processes *etc* (Li et al., 2014). Evidence shows that the disruption of gene expression in the maize endosperm is likely to result in a developmental defection and lead to the failure of seed maturation, possibly due to an inability to provide nutrients and signals for embryo growth. For example, the mutation of *miniature1*, encoding an endosperm-specific isoform of cell wall invertase, reduces kernel fresh weight by approximately 70% by negatively controlling sink strength (Cheng et al., 1996). In addition, the misexpression of the endosperm-specific gene *Meg1* results in malformed transfer cells, which in turn adversely regulates nutrient uptake, sucrose partitioning, and finally seed biomass yield (Costa et al., 2012). These findings indicate precise that the regulation of gene expression in endosperm is required to coordinate proper seed development (Berger et al., 2006).

MiRNAs, which range from 20 to 24 nucleotides (nt) in length, negatively control gene expression through transcript cleavage or translation inhibition through near-perfect complementarity to their targets (Voinnet, 2009). With the advent of high-throughput sequencing technologies, diverse sets of miRNAs have been identified in different plant species on a genome-wide level, exhibiting tissue-specific or/and development-dependent expression patterns (Xue et al., 2009; Jiao et al., 2011; Sun et al., 2014), and our understanding of the functions of these regulatory molecules has also been greatly improved. Various miRNAs have been shown to regulate genes involved in cell differentiation, hormone signaling, stress responses, and organ development, including seeds (Mallory and Vaucheret, 2006). In wheat, 605 miRNAs were identified from developing grains at 5, 15, 25, and 30 DAP. Compared to miRNA abundance in 5-DAP kernels, 114, 147, and 211 miRNAs were found to be significantly up-regulated at the later stages, respectively, among which 86 were potentially associated with the control of grain-filling (Meng et al., 2013). In rice, Zhu et al. (2008) identified 59 miRNA families in two seed developmental stages of japonica species,—0–5 DAP and 6–10 DAPand found that their expression levels changed dynamically, with the highest expression ratio being 18.4:1 (6–10/0–5 DAP), which was observedfor miR408 (Zhu et al., 2008). Moreover, seven out of 36 miRNAs, including osa-miR167, osa-miR397, osa-miR398, osa-miR408, osa-miR528, osa-miR1866-3p, and osa-miRc11 were also preferentially expressed in rice seeds according to miRNA chip data in subspecies of *japonica* (Xue et al., 2009). In addition, a total of 112 *indica* miRNA families were discovered in developing grains, although a discrepancy in expression trends was observed in comparison to previous results (Xue et al., 2009; Lan et al., 2012). In maize, Gu et al. discovered 95 conserved miRNAs and 18 novel miRNAs expressed in 4–6- and 7–23-DAP endosperms, and nearly half of the conserved miRNAs (45/95) were shown to have higher expression levels in later stages than in earlier, including miR408 which is similar to the findings reported in rice (Zhu et al., 2008; Gu et al., 2013). Jiao et al. also identified 38 novel miRNAs from mixed maize endosperms harvested at 12, 16, 20, and 24 DAP (Jiao et al., 2011). Furthermore, gene ontology (GO) enrichment analysis demonstrated that the putative targets of these grain-abundant miRNAs could be involved in the regulation of carbohydrate and protein metabolism, transcription, and cellular transport functional groups, suggesting their crucial roles in gene regulatory networks in seed development (Xue et al., 2009; Lan et al., 2012; Gu et al., 2013; Meng et al., 2013).

However, the miRNA expression patterns in the reciprocal developing endosperm and how the gene expression of the filial genomes is regulated by miRNA have not been explored. More interestingly, the proportions of the parental miRNAs contribute to endosperm development remain unknown, although we have recently reported that a subset of siRNAs exhibited imprinted expression patterns in the developing maize endosperm (Xin et al., 2014). In the present study, we performed genome-wide analysis of endosperm-abundant miRNAs in B73 and Mo17 reciprocal kernels at 0, 3, and 5 DAP and in endosperms at 7, 10, 15 DAP, and investigated their dynamic and potentially biased expression patterns by using the small RNA-seq data published in our previous study (Xin et al., 2014). Our data revealed that all 18 newly identified miRNAs were enriched in the developing endosperms compared to the kernels and that they also showed partitioned expression levels in the reciprocal crosses. Additionally, we characterized a group of tandem miRNAs which were generated from a single precursor in maize. Finally, our data demonstrated that *Zma*-miR408-5p exhibited a parent-biased expression pattern based on the SNP information and Cleaved Amplified Polymorphic Sequence (CAPS) experiment, further suggesting that parental genomes might not contribute equally to controlling endosperm development in terms of miRNA-mediated regulation networks.

## Materials and methods

### Identification of novel miRNAs and target prediction

After removing adaptors, reads shorter than 15 nucleotides were discarded, and the rest were parsed into FASTA format. Then, the reads were mapped to the reference genome of ZmB73_RefGen_v3.tar.gz using the miRDeep-P program with default algorithms. Next, flanking sequence of approximate 250 bp were extracted to predict secondary structures, and stem-loop structures with qualifying scores were further processed by the miRDeep core algorithm. Only small RNAs consistent with the requirements for plant miRNAs were retained for further analysis. Finally, to obtain high-confidence miRNAs in maize endosperm, we only kept endosperm abundant small RNAs with “T” at the beginning of the 5' end. Putative targets prediction was performed using the *psRNATarget* interface server with default settings.

### Quantitative RT-PCR validation for mature miRNA

The SYBR® PrimeScript miRNA RT-PCR Kit (Code No. RR716) was used following the manufacturer's instructions (TaKaRa). Briefly, the reverse transcription was performed by using 1 μg of total RNA incubated with 10 μL of 2 × miRNA Reaction Buffer Mix with Universal Adaptor Primer containing Oligo-dT, 2 μL of 0.1% BSA, 2 μL of miRNA PrimeScript RT Enzyme Mix, and 5 μL of RNase Free dH_2_O in a 20-μL reaction mixture. The temperature program was adjusted to run for 60 min at 37°C, 5 s at 85°C, and then 4°C forever, the Universal Adaptor Primer contained the Uni-miR qRT-PCR primer binding site, the universal reverse primer for qRT-PCR of miRNA.

QRT-PCR of miRNA was conducted on a Bio-Rad CFX96TM Real-Time System. Each reaction included 2 μL of product from the diluted RT reactions, 1.0 μL of miRNA primer (10 μM), 12.5 μL of SYBR Premix Ex Taq II(2 ×), and 8.5 μL of RNase Free water. The reactions were incubated in a 96-well plate at 95°C for 30 s, followed by 40 cycles of 95°C for 5 s, 59°C for 30 s, and 72°C for 30 s. All reactions were run in three replicates for each sample. The value of 2^−ΔΔct^ and standard deviation were then calculated from three replicates, and statistical analysis was performed by using *t*-test (*p* < 0.05). The *Zma-actin* gene was used as the endogenous control. All of the primers used in these analyses are listed in Table S1, the Uni-miR qRT-PCR primer binding to Universal Adaptor Primer for the real time PCR reaction was provided by the kit.

### Cleaved amplified polymorphic sequence (CAPS)

Experimental validation of the biased expression patterns of *Zma*-miR408-5p was performed using CAPS assays as previously described (Konieczny and Ausubel, 1993). RT-PCR was performed using the primers listed in Table S1 which is available online. Amplification products were digested with the restriction enzyme *Mcl*uI to differentiate the transcripts derived from the B73 and Mo17 alleles.

## Results

### Global comparison of conserved miRNA profiling reveals their complexity during maize kernel and endosperm development

**Figure 1:**
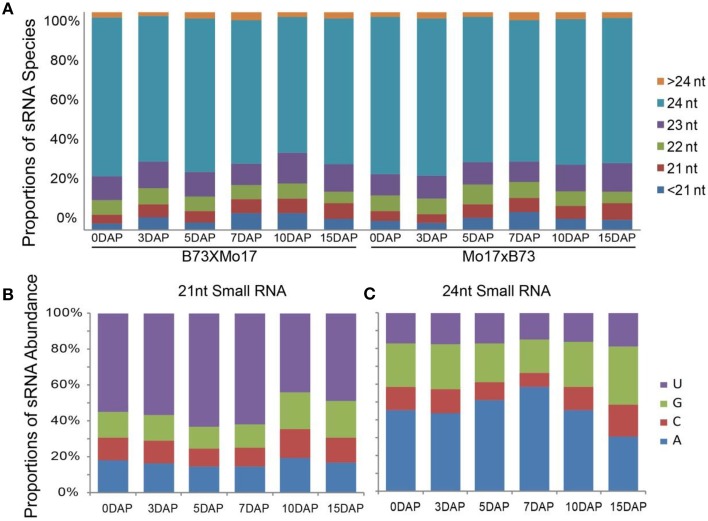
**Distribution features of small RNA species in kernel and endosperm small RNA libraries. (A)** Proportions of distinct small RNA spceies in 12 sequencing libraries. **(B)** Proportions of abundance for 21-nt small RNAs with A, C, G, and U at the beginning of the sequence, during kernel and endosperm developmental stages. **(C)** Proportions of abundance for 24-nt small RNAs with A, C, G, and U at the beginning of the sequenceduring kernel and endosperm developmental stages. DAP, days after pollination.

To study the potential roles of miRNAs in developing maize kernels and endosperms, 12 siRNA libraries of 0-, 3-, and 5-DAP kernels and 7-, 10-, and 15-DAP endosperms from the B73 and Mo17 reciprocal crosses were sequenced (Xin et al., 2014)to observe key events in endosperm development such as cellularization, proliferation, differentiation, and nutrient accumulation (Lopes and Larkins, 1993; Sabelli and Larkins, 2009). The majority siRNAs of our dataset were 24 nt in length and accounted for approximately 68% of total distinct reads, whereas ~12.5% siRNA reads belong to the 21-nt family, this result is similar to those of previous studies (Jiao et al., 2011; Gu et al., 2013) (Figure 1A). Further investigation revealed that a large proportion of 24-nt siRNA reads begin with A at the 5′ end (46.0% average, 29.2 ~ 60.3%), whereas more 21-nt siRNAs begin with T (54.4% average, 40.2 ~ 66.6%) (Figures 1B,C).

**Figure 2:**
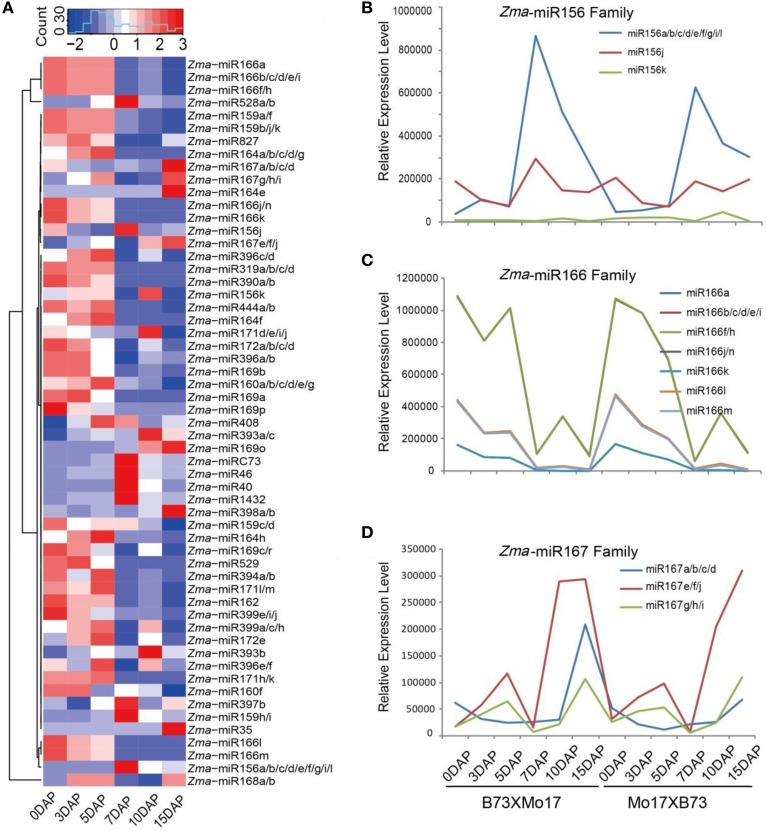
**Conserved miRNA expression pattern during maize kernel and endosperm development in reciprocal crosses. (A)** Hierarchical tandem of 57 conserved miRNAs with relatively high expression levels in at least one developmental stage. Diverse and tissue-specific miRNA expression patterns were exhibited, and a large proportion of miRNAs were highly expressed in kernels whereas only a few miRNAs were abundantly expressed during endosperm stages. **(B)** Dynamic and differential expression patterns of maize miR156 family members. The *Zma*-miR156a group has the highest expression level compared with other family members during all the developmental stages. **(C)** Kernel-abundant expression patterns of maize miR166 family members. Despite different expression levels, *Zma*-miR166 family members showed similar expression trends: they were highly expressed in 0-, 3-, and 5-DAP kernels but not in endosperms. **(D)** Endosperm-abundant expression patterns of maize miR167 family members. All *Zma*-miR167 members exhibited higher expression levels in endosperm stages than in the kernel stages. DAP, days after pollination.

In previous study, we mainly focused on siRNA expression patterns, while here, we paid more attention to miRNA expression trends in the B73 and Mo17 reciprocal endosperms. After removing adaptors and low-quality sequences, the ~163.2 million filtered reads were aligned against mature maize miRNAs registered at miRBase (Release 21), idenfying 57 known miRNA members belonging to 25 families during maize kernel and endosperm developmental stages (Figure 2A). The expression levels of these miRNAs were normalized to the total reads of each library (RPM, Reads Per Million). Among them, *Zma*-miR168, *Zma*-miR166, *Zma*-miR156, *Zma*-miR528, *Zma*-miR827, and *Zma*-miR167 were the top six most abundantly expressed miRNAs in both reciprocal crosses, corresponding to more than 98% of the total number of known miRNAs (Table S2, Figure 2A). As predicted, a proportion of these conserved miRNAs exhibited temporal expression patterns in the kernels and endosperms, e.g., *Zma*-miR166 was predominantly expressed in 0-, 3-, and 5-DAP kernels, on average, and was ~8.7-fold higher than in 7, 10, 15 DAP endosperms. In contrast, the expression level of *Zma*-miR156 was significantly higher in the endosperm stages than in the kernel stages. In addition, *Zma*-miR528 and *Zma*-miR167 showed dramatically varied expression patterns during endosperm development. While the expression of *Zma*-miR528 was significantly down-regulated from 7- to 15-DAP endosperms, a rapid up-regulation of *Zma*-miR167 was observed at 10- and 15-DAP. Although the expression level of *Zma*-miR827 showed a sharp up-regulation at 15 DAP compared with 7 and 10 DAP, it was more highly expressed in maize kernels than in the endosperms. Unlikely, *Zma*-miR168 was consistently expressed with high levels during both kernels and endosperms, possibly because of its key role in regulating the production of miRNAs (Mallory and Vaucheret, 2006) (Figure 2A).

Furthermore, we examined the expression patterns of different members in one miRNA family and observed a expression discrepancy among then. For example, the reads abundance and expression trends of *Zma*-miR156 family are mainly contributed by the member of *Zma*-miR156a, which peaks at 7 DAP and then decreases gradually at later stages, whereas *Zma*-miR156k and *Zma*-miR156j have low and moderate expression levels, respectively, and the latter is expressed during all kernel and endosperm stages with no obvious variation (Figure 2B). Unlike *Zma*-miR156, *Zma*-miR166 family members exhibited similar expression patterns, but their dynamic expression status was likely the results of the transient expression of *Zma*-miR166a/b/f in kernel stages (Figure 2C). In contrast, *Zma*-miR167 family members were all highly expressed in the endosperm stages, although they showed quantitative differences (Figure 2D).

### Identification of novel, temporally expressed miRNAs, and their putative targets during maize kernel and endosperm development

**Figure 3:**
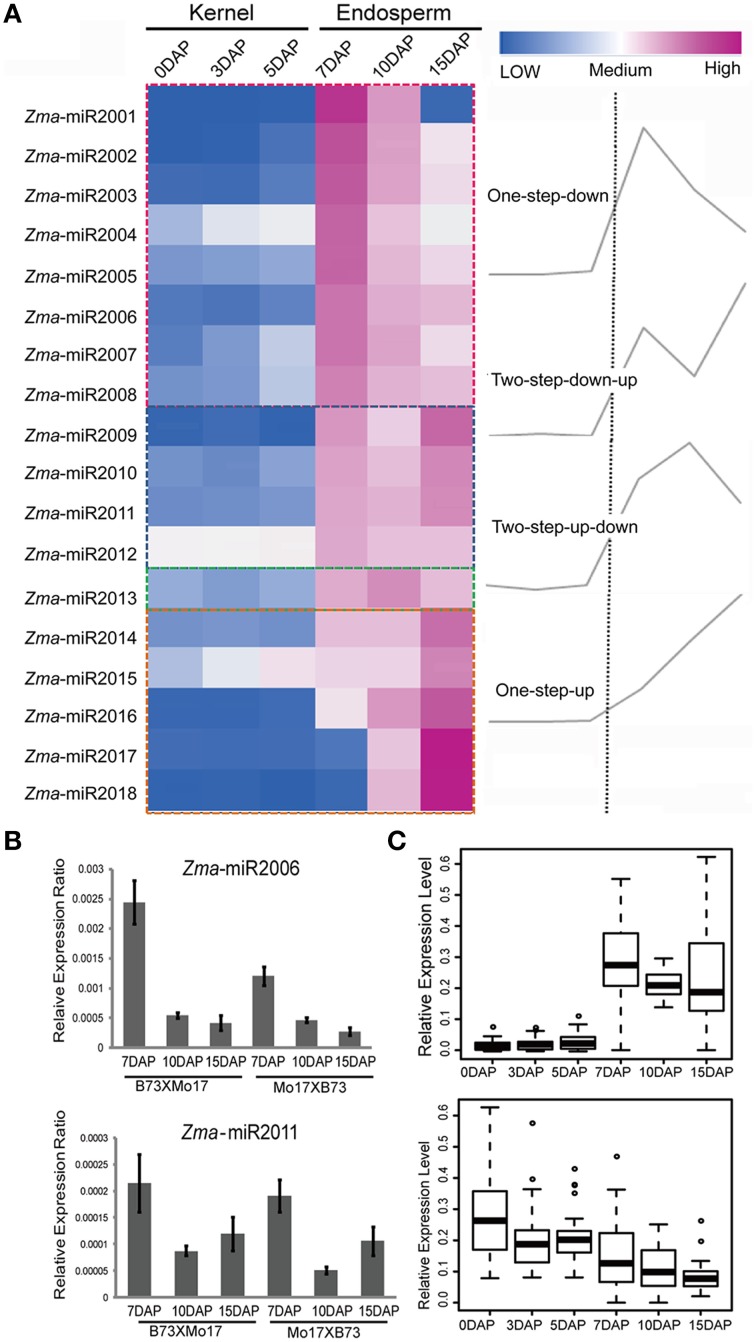
**Developmentally dependent expression patterns of newly identified maize miRNAs negatively correlated with their targets expression. (A)** 18 novel miRNAs were identified in 7-, 10-, and 15-DAP maize endosperms, which can be clustered into four groups according to their expression patterns, namely, one-step-down, one-step-up, two-step-down-up, and two-step-up-down. **(B)** Quantitative RT-PCR results of *Zma*-miR2006 and *Zma*-miR2011. *Zma*-miR2006 showed gradually decreased expression patterns in 7-, 10-, and 15-DAP endosperms in both reciprocal crosses, whereas *Zma*-miR2011 reached its lowest expression level in 10-DAP endosperm. **(C)** Boxplot of expression levels of novel miRNA and their putative targets in kernels and endosperms. MiRNA shows higher abundance in endosperm stages compared to kernel stages; in contrast, their targets exhibited lower expression levels in endosperm stages than in kernel stages, which is consistent with the negative correlation between miRNAs and their targets.

Upon double fertilization, endosperm undergoes a series of biological processes to form the nutritive tissue, which provides nutrients to support embryogenesis and seedling germination. To identify high-confidence miRNAs and investigate their expression profiles during developmental endosperms, we only focused on 21 nt small RNAs with T at the beginning of the 5′ end and reads number exceeding 100 rpm in 12 libraries. Finally, 18 novel putative miRNAs were identified in these six developmental stages based on the mirdeep-p program with the default algorithm (Yang and Li, 2011). Unexpectedly, all 18 miRNAs exhibit significantly increased expression levels in the endosperm compared to the kernels, corresponding to an average of 53.1-fold (2.4 ~ 410.0-fold) higher, although their expression levels among 7-, 10-, and 15-DAP endosperms varied (Table 1). Specifically, after combining the reads amount between two reciprocal crosses, four temporal gene-expression patterns were identified according to their expression trends in endosperms that we described as one-step-up, one-step-down (miRNA level transitions from low to high or high to low, respectively, in the consecutive 7-, 10-, 15-DAP stages), two-step-up-down or two step-down-up (miRNA level transitions from low to high and back down or from high to low and back up, respectively, in a series of endosperm developmental stages). The majority of newly identified miRNAs (14, 77.8%) exhibited a single transition point, of which 27.8% showed the one-step-up pattern, whereas 50% exhibited the one-step down pattern. In contrast, only 22.2% of miRNAs reached their expression peak or trough at 10 DAP (two-step-up-down or two step-down-up) (Figure 3A). Of these newly identified maize miRNAs, the most enriched is *Zma*-miR2001, whose abundance peaks at 7 DAP and then declines at 10 and 15 DAP; overall, its expression level in endosperms is significantly higher than in kernels by approximately 410.0-fold (Figure 3A). To validate the expression patterns of the newly identified miRNAs, quantitative RT-PCR were performed on mature miRNAs of *Zma*-miR2006 and *Zma*-miR2011, and their expression profiles were similar to the results obtained from RNA-seq data (Figure 3B).

**Table 1:** **New identified miRNAs and their putative targets**.

**ID**	**Seq**	**Length (nt)**	**Putative targets**
*Zma*-miR2001	TCTTTTTATTAGTCGTTGGAT	21	GRMZM2G141735,GRMZM2G147014,GRMZM2G551338
*Zma*-miR2002	TGATATCACATGTAGAGGCTG	21	GRMZM2G048022,GRMZM2G108149,GRMZM2G396825
*Zma*-miR2003	TGGAGGGAATTGAAGGGGCTA	21	GRMZM2G029879,GRMZM2G042295,GRMZM2G083374 GRMZM2G098331,GRMZM5G820529,GRMZM2G061620
*Zma*-miR2004	TTGAAGGGGATTGGAGAGGAT	21	GRMZM2G001033,GRMZM2G040642,GRMZM2G134753 GRMZM2G160041,GRMZM2G314647,GRMZM5G894109,AC213619.3
*Zma*-miR2005	TGAGGGGATTGAAGGAGCTAA	21	GRMZM2G005229,GRMZM2G047949,GRMZM2G078396 GRMZM2G098331,GRMZM2G145752,GRMZM2G333659
*Zma*-miR2006	TTCTTAGGAAAAGAGGTCGGC	21	GRMZM2G061620,GRMZM2G065953,GRMZM2G475170,GRMZM5G826456
*Zma*-miR2007	TGGAGAGGATTGTAGGGGCTA	21	GRMZM2G004487,GRMZM2G049660,GRMZM2G085670, GRMZM2G108723,GRMZM2G117677,GRMZM2G136025, GRMZM2G141774,GRMZM2G159709,GRMZM2G179217
*Zma*-miR2008	TTTGGATTGAATTGGTTGGTG	21	GRMZM2G033135,GRMZM2G093436,GRMZM2G100246, GRMZM2G115280,GRMZM2G141273,GRMZM2G401436, GRMZM2G406101,GRMZM5G872216,GRMZM5G889790,AC199705.3
*Zma*-miR2009	TTGGATTTTGATTGGATGCAC	21	GRMZM2G017868,GRMZM2G051090,GRMZM2G053958, GRMZM2G078924,GRMZM2G126197,GRMZM2G133421, GRMZM2G134430,GRMZM2G136311,GRMZM2G312438, GRMZM2G351990,GRMZM2G451254,GRMZM5G862565
*Zma*-miR2010	TGGAGGGGATTGGAGAGGCTA	21	GRMZM2G040642,GRMZM2G072614,GRMZM2G083374, GRMZM2G098331,GRMZM2G314647
*Zma*-miR2011	TCTAAAATGAGTGGTGCTGAT	21	GRMZM2G031572,GRMZM2G082792,GRMZM2G091419, GRMZM2G108023,GRMZM2G127230,GRMZM2G156877
*Zma*-miR2012	TTTGGATCTAGAATCAAAGGC	21	GRMZM2G027522,GRMZM2G034362,GRMZM2G100121, GRMZM2G171430,GRMZM2G172584,GRMZM2G441768
*Zma*-miR2013	TAATTTAGGGACTAAAATGGA	21	GRMZM2G026962,GRMZM2G061396,GRMZM2G082362, GRMZM2G141121,GRMZM2G381744,GRMZM2G399320
*Zma*-miR2014	TGAAGAGAATTGAGGGGGCTA	21	AC155434.2
*Zma*-miR2015	TGTGGATTAGGTGGGATTGGA	21	GRMZM5G884280,GRMZM2G408598,GRMZM2G145916, GRMZM2G099891,GRMZM2G088549,GRMZM2G024104,GRMZM2G010551
*Zma*-miR2016	TGAGGAGTCAAGTAGAACGGA	21	GRMZM2G471335,GRMZM2G363893,GRMZM2G092607, GRMZM2G047486,GRMZM2G032640,
*Zma*-miR2017	TCGTTTTAGTTGTCGTTGGAT	21	
*Zma*-miR2018	TCTCGAAGCGAGTCTGAGTGA	21	GRMZM2G336065,GRMZM2G044398

Subsequently, we predicted the putative targets of these abundantly endosperm-expressed miRNAs by running the *psRNATarget* program with default settings (http://plantgrn.noble.org/psRNATarget/?function=1). In total, 105 potential miRNA target genes were identified. To further confirm the correlation between miRNAs and target genes, we examined the expression patterns of these putative targets in our previous RNA-Seq data obtained in parallel using the same materials. Forty-seven out of 105 genes were found to be expressed (FPKM ≥ 1) in at least one of the developmental stages (Table S3), and interestingly, more than 80% of the target genes showed a higher expression level in the kernels than in endosperms. Further statistical analysis indicated that endosperm-abundant miRNAs have a significant negative correlation with their putative targets (*r* = −0.47, *p* = 8.15E-06) (Figure 3C).

Endosperm cell proliferation occurs at approximately 6–8 DAP (Sabelli and Larkins, 2009), suggesting that miRNAs with an expression pattern of one-step-down at 7 DAP are likely associated with cell cycle regulation. Accordingly, *cyclin p4;1* (GRMZM2G029879) and *cyclin-dependent kinase B2;1* (GRMZM2G141774) were found to be targeted by *Zma*-miR2003 and *Zma*-miR2007, respectively, which reached their peak expression levels in 7-DAP endosperm. Programmed cell death in the starch endosperm facilitates nutrient hydrolysis and uptake by the embryo at germination and begins at approximately 16 DAP in maize (Sabelli and Larkins, 2009). Consistently, *senescence-related gene 1* (GRMZM2G363893) was potentially regulated by *Zma*-miR2016, which exhibited a one-step-up pattern in the 15-DAP endosperm. Interestingly, one of the putative targets of *Zma*-miR2010 is GRMZM2G083374, which encods a protein of unknown function but for which the mutation of its homolog in *Arabidopsis* caused a defective embryo. Therefore, although the exact biological functions of these miRNAs in the endosperm remain ambiguous, our results indicate that the dynamic activity and temporal expression patterns of miRNAs might play a role in the regulation of the maize endosperm or even embryo development.

### MiRNAs contribute unequally between reciprocal crosses during maize endosperm development

**Figure 4:**
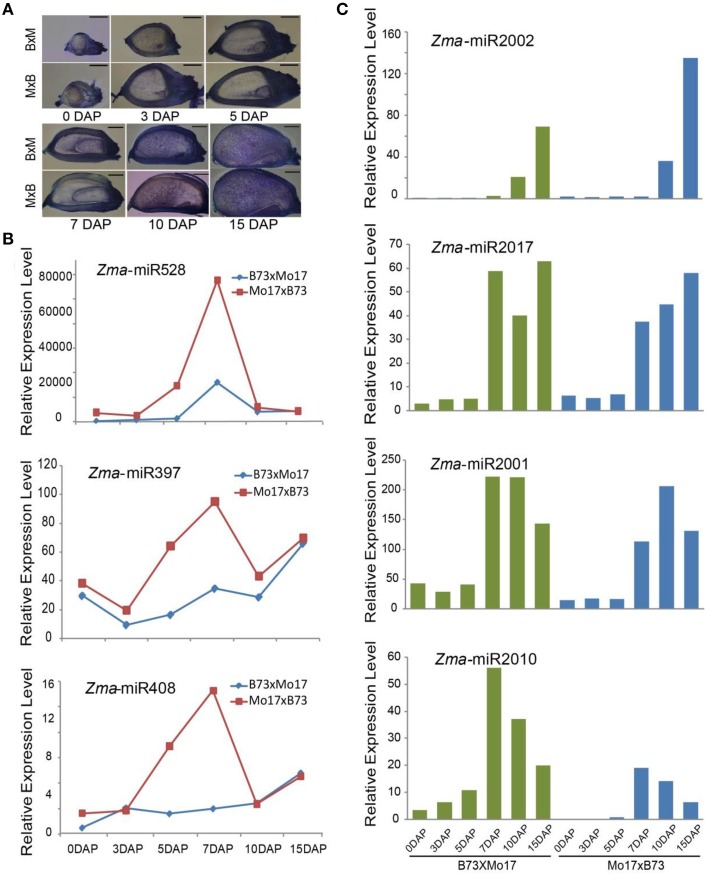
**Phenotypic difference and temporal expression patterns of miRNAs in 0-, 3-, 5-DAP kernels and 7-, 10-, 15-DAP endosperms between B73 and Mo17 reciprocal crosses. (A)** The kernel and endosperm of Mo17 × B73 grow faster than in B73 × Mo17 at all six stages. **(B)** *Zma*-miR528, *Zma*-miR397, and *Zma*-miR408 were more highly expressed in Mo17 × B73 compared to B73 × Mo17 in 5-DAP kernel and 7-DAP endosperm. **(C)** Newly identified *Zma*-miR2002, *Zma*-miR2017, *Zma*-miR2001, and *Zma*-miR2010 exhibited differential expression patterns between reciprocal crosses.

Parental alleles are immediately activated in their filial genomes after fertilization, and their gene expression pattern, level, and timing are undoubtedly associated with plant development (Xin et al., 2013; Li et al., 2014). However, how miRNAs are expressed between reciprocal crosses during maize endosperm development remains undetermined. Our cytological analysis revealed that the endosperm in Mo17 × B73 undergoes faster development than that in B73 × Mo17 (Figure 4A), which led us to determine whether there are differences in the miRNA expression patterns between them. Not surprisingly, a proportion of miRNAs exhibited partitioned expression levels during kernel and endosperm developmental stages between B73 × Mo17 and Mo17 × B73, including both conserved and novel ones. For example, the expression levels of *Zma*-miR528 and *Zma*-miR397 were clearly higher in the Mo17 × B73 cross than in B73 × Mo17 in 5-DAP kernels and 7-DAP endosperms (Figure 4B). Similarly, *Zma*-miR408 also exhibited similar expression trends to *Zma*-miR528 and *Zma*-miR397, which might contribute to the phenotypic difference between these two reciprocal crosses (Figure 4B). It has been predicted that one target of miR528 and miR397 is the *L-ascorbate oxidases* (*AO*) gene, which would increase the accumulation of *dehydroascorbate* (*DHA*) and in turn restrict cell-division ability (Potters et al., 2000; Pignocchi et al., 2006). Therefore, a high abundance of miR397 and miR528 would directly repress the expression of *AO* and indirectly repress that of *DHA*, maintain a high rate of cell division, and thereby promote plant growth. Furthermore, the over-expression of miR408 promotes vegetative growth, but the knockdown of miR408 results in impaired growth in *Arabidopsis*; both of these trends are consistent with the rapid development of Mo17 × B73 kernels and endosperms (Zhang and Li, 2013).

Similarly, although the expression level of *Zma*-miR2002 was predominantly expressed in the late stage of endosperms in both reciprocal crosses, it was approximately 2-fold higher in the Mo17 × B73 cross compared to B73 × Mo17 (Figure 4C). Moreover, *Zma*-miR2017 was barely expressed in all developing kernels, and its expression level exhibited a trough in the 10-DAP endosperm of the B73 × Mo17 cross but a gradual increase from 7 to 15 DAP in the Mo17 × B73 cross (Figure 4C). By contrast, we observed a peak expression level of *Zma*-miR2001 in the 10-DAP endosperm in the Mo17 × B73 cross but no significant change in the Mo17 × B73 cross (Figure 4C). In addition, several newly identified miRNAs were found to be expressed in kernels of one cross but not the other, including *Zma*-miR2010, *Zma*-miR2015, *Zma*-miR2016, and *Zma*-miR2018. Furthermore, the expression level of *Zma*-miR2010 exhibited a sharper decrease in B73 × Mo17 than in Mo17 × B73 (Figure 4C). Taken together, these results indicate that miRNAs might contribute to the phenotypic differences between reciprocal crosses in maize kernel and endosperm development.

### Dynamic expression of tandem miRNAs generated from a common hairpin structure during reciprocal maize endosperm development

**Figure 5:**
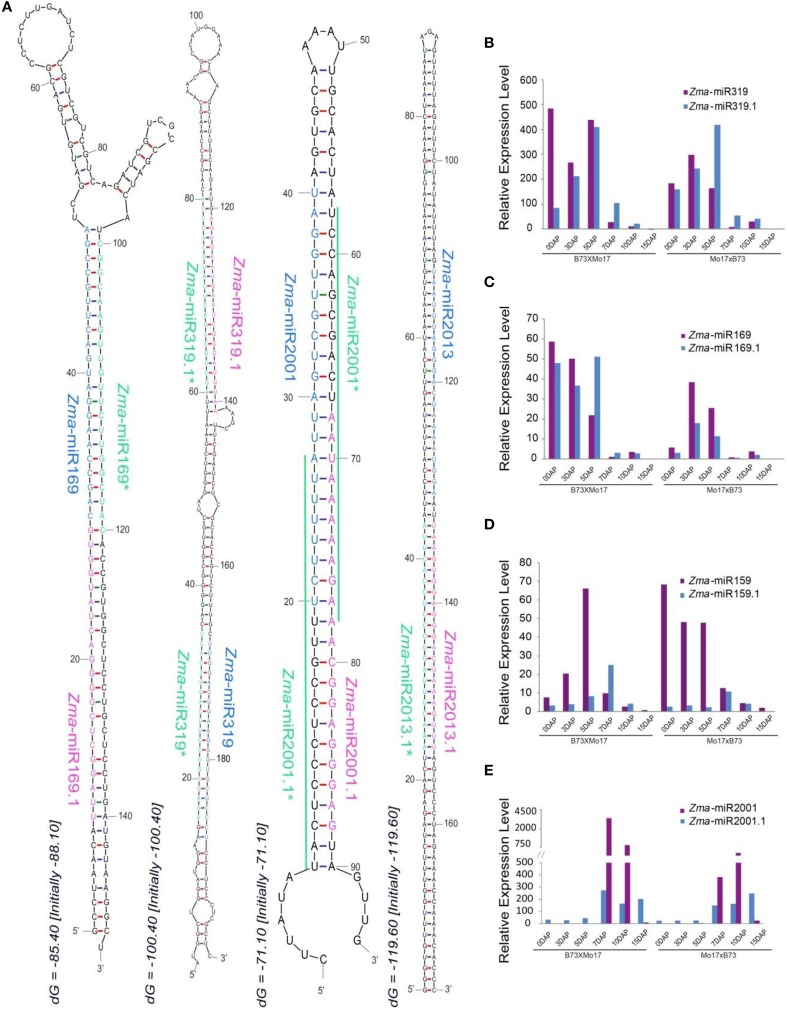
**Biosynthesis and dynamic expression patterns of tandem miRNAs. (A)** Stem-loop structures of tandem miRNAs. *Zma*-miR169&*Zma*-miR169.1, *Zma*-miR319 and *Zma*-miR319.1, *Zma*-miR2001 and *Zma*-miR2001.1, *Zma*-miR2013 and *Zma*-miR2013.1 were generated from a single precursor. **(B)** Varied expression patterns of *Zma*-miR319 and *Zma*-miR319.1 during kernel and endosperm developmental stages. **(C)** Dynamically changed expression patterns of *Zma*-miR169 and *Zma*-miR169.1 during kernel and endosperm developmental stages. **(D)** Temporal expression patterns of *Zma*-miR169 and *Zma*-miR169.1 during kernel and endosperm developmental stages. **(E)** Development-dependent expression patterns of *Zma*-miR2001 and *Zma*-miR2001.1 during kernel and endosperm developmental stages.

Recent analyses of massive amounts of data from high-throughput sequencing have identified subclasses of miRNA species derived from alternative biogenesis pathways except for the canonical miRNAs, including NAT-miRNA, miRtron, snoRNA-derived, and tRNA-derived miRNA (Miyoshi et al., 2010). In this study, we identified a group of miRNAs termed tandem miRNAs which were co-generated from a single hairpin locus. For example, in addition to *Zma*-miR319 and *Zma*-miR319^*^, their precursor also produces another pair of miRNA-like that were closer to the loop structure compared to *Zma*-miR319, these are defined as *Zma*-miR319.1 and *Zma*-miR319.1^*^, respectively (Figure 5A). Similarly, the precursor of *Zma*-miR169 also yields an accompanying miRNA (*Zma*-miR169.1), although its *Zma*-miR169.1^*^ was not detected, possibly due to a relatively low expression level (Figure 5A). In addition, of the newly identified maize miRNAs, *Zma*-miR2001.1, and *Zma*-miR2013.1 were also detected during developing maize endosperm (Figure 5A). Interestingly, the precursors of five members of the miR159 family all exhibited this tandem miRNA expression pattern including *Zma*-miR159a, *Zma*-miR159b, *Zma*-miR159f, and *Zma*-miR159j/k (Figure S1A). Moreover, a previous study has reported that tandem miR159.1 and miR159.2 were co-produced in rice and its relatives (Lacombe et al., 2008), which raised an intriguing question of their evolution in plant species. Thus, we next examined the conservation of miR159 precursors among maize, rice, wheat, and barley, and surprisingly, that they all produced tandem miRNAs, although with several mismatches in miR159.1 among different species (Figure S1B). To summarize, tandem miRNA expression manner is evolutionarily conserved at least in monocots.

Further investigation indicated that these tandem miRNAs also exhibited altered expression patterns during kernel and endosperm development, e.g., both *Zma*-miR319.1 and *Zma*-miR169.1 were predominantly expressed in 0, 3, and 5 DAP kernels, whereas *Zma*-miR159.1 and *Zma*-miR2001.1 were preferentially expressed in endosperms (Figures 5B–E). In addition, these tandem miRNAs showed differential expression trends compared with their “neighbors” during the kernel and endosperm developmental stages in both reciprocal crosses, although they were located in a single hairpin locus. For example, the expression level of *Zma*-miR319.1 gradually increased in 0-, 3-, and 5-DAP kernels, unlike the expression pattern of *Zma*-miR319. In contrast to the observation in the kernels, *Zma*-miR319.1 exhibited a higher expression level in developing endosperms than that of *Zma*-miR319 (Figure 5B). Surprisingly, although the expression level of *Zma*-miR159 was clearly higher in kernels than in endosperms, *Zma*-miR159.1 exhibited the opposite expression patterns: its average expression level was higher in endosperms compared to kernels (Figure 5D). Moreover, the expression abundance of *Zma*-miR2001 was significantly enriched in 7- and 10-DAP endosperms compared with *Zma*-miR2001.1 in both reciprocal crosses; however, the expression patterns were reversed in 15-DAP endosperms (Figure 5E).

### Parentally biased expression of Zma-miR408-5p in maize reciprocal crosses

**Figure 6:**
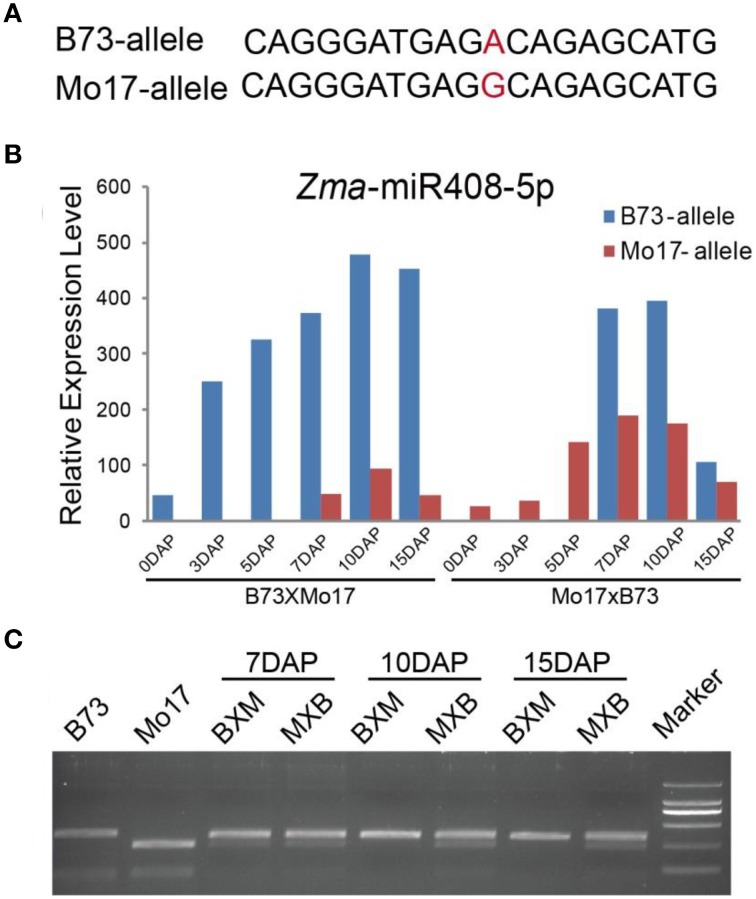
**Biased expression pattern of *Zma*-miR408-5p in 7-, 10-, and 15-DAP endosperms in B73 and Mo17 reciprocal crosses. (A)** SNP of *Zma*-miR408-5p between B73 and Mo17 confirmed by re-sequencing. **(B)** *Zma*-miR408-5p of B73 was more highly expressed compared to that of Mo17 during endosperm developmental stages in both reciprocal crosses based on the sequencing data. **(C)** Validation of biased expression pattern of *Zma*-miR408-5p using CAPS in 7, 10, and 15 DAP endosperms of reciprocal crosses.

A proportion of small interfering RNAs exhibited possible parent-biased expression patterns in the developing plant endosperm between B73 and Mo17 reciprocal crosses (Xin et al., 2014), leading us to investigate whether miRNAs also showed allele-biased expression patterns. We first examined the SNPs located in the endosperm-abundant miRNAs between B73 and Mo17 cultivars. However, no informative SNPs were identified in these small regulatory molecules between the two inbred lines, probably because of their high evolutionary conservation, except for *Zma*-miR408^*^. Further comparison found *Zma*-miR408^*^ was more abundantly expressed than *Zma*-miR408 in both kernels and endosperms, consistent with previous reports (Jiao et al., 2011; Gu et al., 2013) and indicating *Zma*-miR408^*^ probably also has a biological relevance rather than the useless, degraded sequences. Therefore, we designed the original *Zma*-miR408 and *Zma*-miR408^*^ to be *Zma*-miR408-3p and *Zma*-miR408-5p, respectively, based on 3′- or 5′-arm derivation of the miRNA species. Although the well-known conservation of miRNA, a SNP (A/G) between B73 and Mo17 inbred lines in mature *Zma*-miR408-5p sequence was identified and confirmed by sequencing (Figure 6A). Based on the SNP information, we next examined whether this miRNA is subjected to allele-biased expression between the two reciprocal crosses. Interestingly, *Zma*-miR408-5p showed a significantly B73-allele biased expression pattern in 7-, 10-, and 15-DAP reciprocal endosperms (Figure 6B, χ^2^-test, *p*-value ≤ 0.01), which was further confirmed by CAPS experiments (Figure 6C). Moreover, the SNP of *Zma*-miR408-5p was located at the 11th base position from the 5′ end between B73 and Mo17, indicating that this polymorphism was likely to cause functional variation of *Zma*-miR408-5p. Thus, these two should target complete different genes based on the principle of complementary base pairing because the 10/11 base position is very important for miRNA-directed mRNA cleavage (Figure 6B). The target prediction of B73 and Mo17 *Zma*-miR408-5p by the *psRNATarget* server is also consistent with our speculation. The putative targets of B73 *Zma*-miR408-5p were GRMZM2G034896, GRMZM2G093526, and GRMZM2G177518, while Mo17 *Zma*-miR408-5p likely negatively modulates five other targets, including AC196575.3_FG007, GRMZM2G169005, GRMZM2G162065, AC198481.3_FG004, and GRMZM2G349554.

## Discussion

### Complicated miRNA interactions were involved in the regulation of maize endosperm development

MiRNAs are key regulators of gene expression in plants, especially for transcription factors, which gives these small molecules a central role of plant phase transition, organ architecture, and response to stress. Furthermore, recent studies have revealed that different miRNAs are likely to be integrated into functional networks in cellular and physiological events during plant development. One of the most well understood examples is the antagonistic interplay of miR156 and miR172 node, that is, *SPL9*, the down-regulated target of miR156, is able to directly bind to the promoter of miR172b gene and correspondingly promote its expression, which coordinately regulated the transition from vegetative phase to reproductive phase. Specifically, increased abundance of miR156 prolongs the juvenile stage and delays the adult stage transition, while highly expressed miR172 leads to early flowering and premature features of vegetative growth. In addition, miR390 targets a non-protein coding gene and triggers the production of *TAS3*, and as a consequence, which in turn causes the degradation of *AUXIN RESPONSE FACTOR3* (*ARF3*) and *ARF4* mRNAs, and finally advance the process of precocious maturation.

Although these miRNA regulation networks were previously observed in *Arabidopsis*, these interplay nodes might also be applicable during maize kernel and endosperm development according to the miRNA expression profile determined here. For example, miR156 reaches its peak expression at 7 DAP, while miR172 exhibits its lowest expression level at the same developmental stage (Figure S2A). The biological significance underlying this observation is reasonable because the endosperm has completed cell differentiation and formed four compartments at 7 DAP (including basal endosperm transfer cell, aleurone, starch endosperm, and embryo surrounding region), and starch accumulation and storage protein are ready to begin, indicating the end of the “juvenile stage” and ready for “adult stage.” Correspondingly, the abundance of miR156 decreases at 10 and15 DAP while miR172 increases, consistent with the expression trends during leaf aging. Additionally, the miR390-*TAS3* node might also play a role in the regulation of endosperm maturation process, as both were highly expressed in 0-, 3-, and 5-DAP kernels but barely expressed in 7-, 10-, and 15-DAP endosperms. This differential expression pattern would elevate the level of *ARF3*, a target of *TAS3*, and promote precocious entrance to the adult stage (Figure S2B). However, more work is needed to validate these miRNA functions in maize endosperms in the future.

### Multiple pathways exist to generate miRNAs in plants

In flowering plants, the classical mature miRNAs are typically derived from pri-miRNAs transcribed by RNA polymerase II, which is then processed into miRNA precursor and form a stem-loop structure (Kim, 2005). However, a recent study identified two special classes of miRNAs, namely, mirtron and NAT-miRNA, which are originating from splicing-dependent introns of the host genes and the overlapping transcript antisense to the target gene, respectively(Lu et al., 2008; Zhu et al., 2008). In addition, in several cases, tandem stem-loop structures have been reported to be produced within a common precursor in rice, maize, *Medicago truncatula*, and soybean (Guddeti et al., 2005; Chuck et al., 2007; Wang et al., 2007; Zhang et al., 2008). However, in this study, we confirmed that tandem but distinct miRNAs can be generated from a single transcribed locus in maize and verified that this pathway of producing miRNA could be conserved in monocot plants. This seems to be a more efficient way to produce miRNAs in terms of energy consumption, similar to the production of siRNAs. In previous study, it has been reported that miR159.1 and miR159.2 were simultaneously generated from a single precursor in rice (Lacombe et al., 2008), however, we found this phenomena was not a unique case and expression of tandem miRNAs could represent a new mechanism to produce multiple miRNAs in monocot plants. More interestingly, although co-transcribed from a common precursor, tandem miRNAs are not expressed completely synchronously; instead, they sometimes exhibit different, even antagonistic expression trends during maize kernel and endosperm development (Figure 5), indicating the production of these tandem miRNAs might be controlled at multi-levels, e.g., transcription of the pri-miRNA during any of the biogenesis steps, or at turnover of the mature miRNA. However, more efforts are needed to validate our prediction of these potential miRNAs, especially the confirmation of their biosynthesis in *dcl1* maize mutants.

### A subset of miRNAs probably exhibited allele-biased expression patterns in maize endosperm

Allele-biased expression occurs to small interfering RNAs in the endosperm of *Arabidopsis*, rice, and maize and their expression patterns are subjected to dynamic changes during endosperm development (Mosher et al., 2009; Rodrigues et al., 2013; Xin et al., 2013). Further analysis of these siRNAs with allele-biased expression in *Arabidopsis* indicates that they likely influence the expression of *AGAMOUS-LIKE* genes and result in small seeds, leading us to examine whether miRNAs exhibit similar expression patterns (Lu et al., 2012).

Unfortunately, because of the high conservation of sequence, SNP information is unavailable for allele-biased expression analysis of most miRNAs except for *Zma*-miR408-5p, which contains an A/G transition at the 11th position from the 5′ end of the sequence. Based on the reads count in deep sequencing data, the expression of *Zma*-miR408-5p is B73 allele-biased. In addition, CAPS validation of the miRNA408 precursor further confirmed our hypothesis. More interestingly, the SNP variation in *Zma*-miR408-5p between B73 and Mo17 would cause unexpected target changes. In this scenario, a subset of genes (e.g., targets for Mo17 *Zma*-miR408-5p), which will not be down-regulated in inbred lines (e.g., B73), would be unavoidably repressed after hybridization, and *vice versa*. This regulation pattern maybe fit dominant model to partially explain heterosis in F1, and their distinct expression profile during kernel and endosperm development in the reciprocal crosses might be associated with phenotypic difference between each other. However, we cannot excluded the possibility that Zma-miR408^*^ of B73 is a star miRNA that might be more abundantly expressed or just more stable compared to that of Mo17 without further biological validation. In conclusion, our study proved that not only protein coding gene and small interfering RNA but also a proportion of miRNAs might exhibit allele-biased expression pattern, but their biosynthesis and biological relevance need further investigation.

## Author contributions

ZN, XW, QS conceived the project. MX and GY collected the plant materials. MX, YY, HP, and ZH analyzed data. MX, XW, and NZ wrote the manuscript.

## Conflict of interest statement

The authors declare that the research was conducted in the absence of any commercial or financial relationships that could be construed as a potential conflict of interest.

## Supplementary material

Table S1 **The primer pairs for miRNA qRT-PCR**.

Table S2 **The expression level of conserved miRNAs in 0-, 3-, 5-DAP kernels and 7-, 10-, 15-DAP endosperms of B73 and Mo17reciprocal crosses (RPM: reads per million)**.

Table S3 **The expression pattern of putative targets of 47 miRNA in 0-, 3-, 5-DAP kernels and 7-, 10-, and 15-DAP endosperm in B73 and Mo17 reciprocal crosses**.

Figure S1 **The stem-loop structures of precursors of miR159 and their tandem miRNAs in maize (A) and the sequences of miR159 in maize, wheat, barley, and rice (B)**.

Figure S2 **The expression pattern of miR156 and miR172 (A) as well as miR390 and TAS3 (B) in 0-, 3-, 5-DAP kernels and 7-, 10-, and 15-DAP endosperm in maize**. RPM, reads per million.

## Abbreviations

DAP: days after pollination
CAPS: Cleaved Amplified Polymorphic Sequence
nt: nucleotide
*ARF*: *AUXIN RESPONSE FACTOR3*
*AO*: *L-ascorbate oxidases*
*DHA*: *dehydroascorbate*
GO: gene ontology
RPM: reads per million.
