# Supplementary material for: Temporal small RNA transcriptome profiling unraveled partitioned miRNA expression in developing maize endosperms between reciprocal crosses
Source: Front Plant Sci. 2015 Sep 15;6:744. doi: 10.3389/fpls.2015.00744 (PMC4584948; doi:10.3389/fpls.2015.00744)
Supplement: Table S1 — The primer pairs for miRNA qRT-PCR. [file DataSheet1.PDF]

Table S1 Primer pairs used for miRNA qRT-PCR

Primer namer	Primer sequence
Zma-miR2006-R	TTCTTAGGAAAAGAGGTCGGC
Zma-miR2011-R	TCTAAAATGAGTGGTGCTGAT
Zma-actin-L	CGATTGAGCATGGCATTGTCA
Zma-actin-R	CCCACTAGCGTACAACGAA

Table S2. The expression level of conserved miRNAs in 0-,3-,5-DAP kernels and 7-,10-,15-DAP endosperms of B73 and Mo17reciprocal crosses (RPM: reads per million)

ID	BM0DAP	BM3DAP	BM5DAP	BM7DAP	BM10DAP	BM15DAP	MB0DAP	MB3DAP	MB5DAP	MB7DAP	MB10DAP	MB15DAP
zma-miR1432	6.06	13.34	12.79	421.31	70.55	21.78	4.09	6.76	6.89	65.77	21.70	11.04
zma-miR156a/b/c/d/e/f/g/i/l	1793.79	5628.83	4214.56	51928.26	23579.03	12086.45	3102.47	3727.46	5400.91	46068.05	15059.01	12562.40
zma-miR156j	8966.66	5244.52	4437.72	17664.62	5190.58	5768.91	13473.07	5798.36	4930.19	13745.03	5831.07	8191.46
zma-miR156k	164.65	299.64	252.18	94.18	431.16	19.08	949.38	1223.28	1333.85	143.75	1918.38	19.75
zma-miR159a/f	1553.05	2636.09	2227.35	554.20	263.83	24.12	4010.99	2209.65	2650.70	690.98	241.95	38.27
zma-miR159b/j/k	1548.51	2617.89	2214.55	549.71	261.44	23.88	3989.30	2194.50	2632.71	687.43	239.90	38.15
zma-miR159c/d	7.19	8.49	12.06	17.86	2.19	0.00	26.72	12.44	13.21	7.03	14.94	0.12
zma-miR159h/i	0.00	0.00	1.72	35.71	9.55	0.74	0.00	0.00	1.26	22.79	6.75	2.58
zma-miR160a/b/c/d/e/g	82.52	35.67	83.90	18.67	34.62	0.74	5.66	62.21	64.80	26.26	6.14	0.12
zma-miR160f	0.76	0.49	0.00	0.41	0.40	0.00	0.00	0.27	0.28	0.07	0.00	0.00
zma-miR162	4.92	3.40	6.15	0.41	0.99	0.62	12.26	9.20	6.18	1.18	2.87	0.12
zma-miR164a/b/c/d/g	440.97	3598.33	3560.36	250.66	171.11	16.74	5230.41	5414.31	8751.84	538.51	717.65	37.54
zma-miR164e	10.22	14.07	37.89	648.93	366.09	6502.64	15.40	21.10	58.33	428.65	287.80	7293.04
zma-miR164f	10.60	109.91	91.28	9.54	4.97	0.74	148.07	150.65	219.40	17.83	23.33	1.84
zma-miR164h	1.51	14.56	10.58	1.36	1.59	0.00	16.35	13.52	29.52	2.89	4.09	0.49
zma-miR166a	52090.58	42760.44	60986.94	6472.04	12054.45	4159.43	70240.85	67201.92	50135.27	4546.44	14887.27	4921.36
zma-miR166b/c/d/e/i	52086.80	42755.83	60977.09	6471.22	12053.06	4158.94	70232.68	67194.89	50130.49	4545.84	14885.43	4920.50
zma-miR166f/h	52437.30	42814.06	60992.35	6456.77	12102.21	4070.09	70619.04	67397.19	50453.76	4632.40	15074.98	4840.03
zma-miR166j/n	7724.37	4673.14	5009.75	178.28	88.14	14.03	10955.00	7472.50	5020.85	212.40	245.43	13.62
zma-miR166k	7725.88	4672.90	5009.50	178.42	87.94	14.03	10955.00	7470.61	5019.02	212.47	245.22	13.74
zma-miR166l	21064.02	12613.68	14769.92	1090.42	1084.75	343.48	31154.55	19673.97	14635.13	803.21	1733.13	383.10
zma-miR166m	20784.68	12354.56	14416.37	1051.71	954.43	239.24	30731.10	19299.92	14263.22	776.65	1527.82	287.41
zma-miR167a/b/c/d	3015.26	1672.88	1462.67	1557.12	1061.08	8974.20	3389.17	1461.56	856.53	1582.97	1114.96	2859.17

zma-miR167e/f/j	811.16	2994.44	7031.16	938.30	10266.96	12633.12	2023.26	4960.75	7094.43	502.70	8511.93	12966.35
zma-miR167g/h/i	776.71	2113.72	3877.01	427.99	761.83	4553.62	1707.00	3125.96	3834.02	387.37	987.23	4567.21
zma-miR168a/b	214647.2	284785.6	269030.1	204157.0	166692.93	297057.69	217361.6	292096.8	296717.4	178067.2	199041.7	285452.6
zma-miR169a	114.31	102.14	44.04	2.04	4.78	0.49	49.36	74.65	35.56	1.63	5.94	0.37
zma-miR169b	62.83	52.89	22.64	1.09	3.98	0.25	24.21	39.49	27.55	0.89	4.09	0.12
zma-miR169c/r	13.63	11.89	4.92	0.14	5.97	0.00	17.60	13.52	8.71	0.15	9.83	0.00
zma-miR169o	0.38	0.00	0.00	10.77	69.64	104.61	1.57	0.27	0.56	11.10	62.43	106.60
zma-miR169p	62.83	27.42	22.14	1.77	0.60	0.00	182.96	68.70	30.22	0.67	1.64	0.00
zma-miR171d/e/i/j	92.36	91.95	53.39	28.49	120.17	12.68	81.42	63.29	50.18	55.12	143.49	19.50
zma-miR171h/k	1.14	2.67	2.95	0.00	0.00	0.00	2.83	1.35	1.55	0.07	0.20	0.00
zma-miR171l/m	7.95	5.10	10.33	1.09	0.80	0.00	8.49	7.30	11.81	1.04	1.64	0.00
zma-miR172a/b/c/d	61.70	37.12	18.21	1.50	13.93	14.52	62.87	61.39	44.41	2.52	44.42	5.89
zma-miR172e	1.89	3.64	6.15	0.14	1.59	1.11	2.20	7.30	9.28	0.81	6.75	0.49
zma-miR319a/b/c/d	549.60	315.41	496.50	39.39	14.13	0.37	235.15	344.02	195.23	11.25	36.84	0.12
zma-miR390a/b	415.23	271.25	247.26	73.19	18.11	1.72	1085.19	917.13	687.45	84.49	82.90	4.91
zma-miR393a/c	1.89	17.95	19.93	14.86	39.99	26.46	7.86	15.15	24.46	17.39	59.77	30.67
zma-miR393b	0.00	3.88	3.94	2.59	12.14	3.20	1.89	2.43	5.62	2.96	11.05	4.29
zma-miR394a/b	23.47	1.46	8.37	0.82	4.58	0.00	2.51	10.28	21.93	0.44	0.41	0.00
zma-miR396a/b	26.12	26.69	24.60	4.77	12.73	20.18	71.68	76.27	42.03	3.99	35.62	21.84
zma-miR396c/d	140.81	260.58	358.23	68.97	118.98	34.46	258.09	362.96	355.32	50.83	229.46	50.17
zma-miR396e/f	0.76	1.46	1.72	0.82	1.79	0.74	2.83	1.08	3.09	0.15	1.84	1.35
zma-miR397b	1.14	0.00	0.49	1.77	0.80	2.83	2.20	0.81	4.64	7.40	1.64	2.94
zma-miR398a/b	43.15	25.48	26.82	10.90	67.85	292.90	19.49	17.58	53.27	25.23	49.54	221.79
zma-miR399a/c/h	3.79	6.79	4.67	0.55	3.38	0.49	3.46	3.52	8.01	0.30	6.14	0.49
zma-miR399e/i/j	11.73	10.19	2.71	0.55	0.40	0.00	11.00	3.52	4.64	0.22	0.41	0.00
zma-miR408	9.46	27.90	25.10	16.36	26.06	32.24	26.09	33.27	68.59	67.77	28.66	32.02

zma-miR444a/b	69.27	48.77	107.02	31.49	21.09	21.91	182.96	152.00	146.88	44.76	49.33	31.04
zma-miR528a/b	7005.95	13923.11	23518.40	139734.6	46440.67	30842.91	65316.64	51112.61	151151.8	354710.0	76664.64	32384.60
zma-miR529	10.98	16.74	9.60	1.09	0.80	0.00	16.98	14.06	5.90	0.30	1.43	0.12
zma-miR827	2268.44	2613.52	1740.94	621.13	550.53	1337.13	2170.06	2716.22	2216.95	280.68	529.95	1479.51
miR35	0.13	0.06	0.00	2.48	2.73	46.55	0.00	0.00	0.06	1.98	1.69	17.13
miRC73	1.57	2.74	5.35	65.21	14.60	3.35	1.45	2.83	8.13	75.78	14.54	6.64
miR40	0.13	0.06	0.00	82.38	19.89	0.32	0.07	0.06	0.42	32.14	8.75	0.58
miR46	1.45	2.61	4.29	55.28	8.74	5.18	2.00	2.83	6.08	75.59	9.95	6.54

Table S3. The expression pattern of putative targets of 47 miRNA in 0-, 3-, 5-DAP kernels and 7-, 10- and 15-DAP endosperm in B73 and Mo17 reciprocal crosses

		**B73XMo17**						**Mo17XB73**					
miRNA ID	Gene ID	0DAP	3DAP	5DAP	7DAP	10DAP	15DAP	0DAP	3DAP	5DAP	7DAP	10DAP	15DAP
Zma-miR2014	AC155434.2	0.840	0.311	0.260	0.000	0.000	0.108	0.100	0.894	1.293	0.000	0.049	0.275
Zma-miR2008	AC199705.3	0.000	0.000	0.000	0.000	0.036	0.000	0.000	0.000	0.000	0.000	0.000	0.000
Zma-miR2005	GRMZM2G005229	3.395	4.256	2.973	0.757	0.498	1.083	8.557	7.314	3.027	0.905	1.319	0.765
Zma-miR2011	GRMZM2G031572	25.587	20.801	34.988	19.811	31.433	29.127	29.629	27.959	51.688	19.576	37.178	55.535
Zma-miR2016	GRMZM2G032640	0.171	0.106	0.048	0.000	0.057	0.000	0.408	0.107	0.027	0.000	0.000	0.000
Zma-miR2016	GRMZM2G034526	40.811	7.087	18.071	8.369	6.653	0.755	50.310	24.293	14.612	8.733	8.203	1.431
Zma-miR2004	GRMZM2G040642	6.107	5.026	5.781	7.846	2.777	0.661	10.160	8.347	8.349	7.911	3.510	1.562
Zma-miR2018	GRMZM2G044398	41.157	18.738	32.349	52.876	20.861	6.133	58.766	31.907	23.067	48.596	15.999	8.799
Zma-miR2002	GRMZM2G048022	0.472	3.553	1.771	0.727	0.000	0.000	1.239	3.083	5.419	0.506	0.000	0.000
Zma-miR2009	GRMZM2G053958	2.033	2.370	2.468	5.935	2.028	0.163	5.260	6.062	4.894	8.638	6.557	1.639
Zma-miR2013	GRMZM2G064896	10.165	22.424	11.418	16.891	9.390	1.828	11.184	18.445	16.329	23.976	12.994	4.513
Zma-miR2006	GRMZM2G065953	13.532	15.780	14.083	56.021	26.810	4.993	0.000	0.354	1.470	24.792	9.388	5.290
Zma-miR2010	GRMZM2G072614	0.000	0.000	0.041	0.000	0.012	0.070	0.000	0.000	0.000	0.000	0.000	0.022
Zma-miR2011	GRMZM2G082792	44.754	35.651	13.335	0.736	0.975	0.357	2.787	3.626	4.359	1.832	1.222	0.480
Zma-miR2015	GRMZM2G088549	16.306	7.078	14.683	11.859	13.489	4.824	29.062	19.428	22.095	13.951	19.061	10.977
Zma-miR2008	GRMZM2G093436	14.322	14.097	18.417	19.939	18.667	5.363	16.299	16.436	10.393	20.163	18.970	7.145
Zma-miR2009	GRMZM2G099337	27.988	2.793	23.594	13.321	9.381	3.193	35.020	15.007	14.981	9.427	12.512	7.507
Zma-miR2009	GRMZM2G104401	0.000	0.000	0.000	0.088	0.507	0.146	3.022	0.663	0.809	0.090	0.439	0.000
Zma-miR2002	GRMZM2G108149	87.717	41.528	65.053	11.735	24.180	0.555	83.587	50.167	40.580	12.911	18.625	0.801
Zma-miR2007	GRMZM2G117677	0.000	0.000	0.000	0.021	0.000	0.000	0.000	0.000	0.000	0.000	0.041	0.000
Zma-miR2009	GRMZM2G126197	7.709	4.826	8.298	9.863	5.331	1.954	9.414	8.187	5.856	8.049	7.365	3.368
Zma-miR2011	GRMZM2G127230	1.148	0.262	0.617	0.200	0.084	0.080	2.113	1.100	0.365	0.148	0.036	0.019

Zma-miR2011	GRMZM2G127893	4.953	4.988	4.394	4.021	0.800	0.164	14.413	9.912	7.519	2.572	1.451	0.136
Zma-miR2009	GRMZM2G133421	10.394	10.484	13.175	7.239	5.951	0.742	10.149	11.644	10.570	7.288	8.418	1.185
Zma-miR2007	GRMZM2G136025	0.000	0.000	0.000	0.021	0.000	0.000	0.000	0.000	0.000	0.000	0.000	0.000
Zma-miR2013	GRMZM2G141121	1.939	4.691	1.552	0.000	0.000	0.000	0.000	0.036	0.000	0.000	0.000	0.000
Zma-miR2001	GRMZM2G141735	17.431	5.220	14.183	21.457	5.659	2.125	34.709	21.696	20.712	13.155	11.049	2.017
Zma-miR2007	GRMZM2G141774	0.000	0.000	0.000	0.041	0.000	0.000	0.000	0.000	0.000	0.000	0.000	0.000
Zma-miR2005	GRMZM2G145752	9.591	3.492	9.293	10.441	4.728	0.712	18.203	10.377	12.429	7.674	4.935	2.580
Zma-miR2015	GRMZM2G145916	6.246	0.981	1.786	2.275	0.125	0.000	7.850	2.106	3.992	0.718	0.488	0.891
Zma-miR2001	GRMZM2G147014	83.697	145.260	277.210	5.145	39.410	5.280	195.333	208.979	166.103	7.782	30.232	7.360
Zma-miR2007	GRMZM2G159709	0.000	0.000	0.000	0.000	0.000	0.000	0.000	0.000	0.000	0.000	0.000	0.000
Zma-miR2012	GRMZM2G172584	16.011	18.323	15.031	16.118	24.890	12.366	12.937	10.858	10.953	18.557	21.586	18.014
Zma-miR2009	GRMZM2G178886	7.412	7.142	5.167	6.134	6.522	0.785	5.364	7.577	10.476	8.509	12.088	1.718
Zma-miR2007	GRMZM2G179217	10.014	2.601	7.992	7.369	5.550	2.166	11.727	6.518	9.238	5.945	6.404	1.787
Zma-miR2004	GRMZM2G314647	31.877	11.340	43.792	53.820	25.446	9.626	35.043	29.350	36.475	54.641	40.508	19.410
Zma-miR2018	GRMZM2G336065	45.767	27.324	31.944	28.831	14.146	5.785	63.590	36.626	22.788	28.854	11.274	7.419
Zma-miR2009	GRMZM2G351990	13.138	22.349	14.649	12.288	10.989	1.273	18.147	17.211	22.396	18.231	17.611	1.798
Zma-miR2016	GRMZM2G363893	1.910	7.824	6.398	1.760	0.635	0.137	3.264	6.517	7.420	1.080	2.146	0.325
Zma-miR2013	GRMZM2G381744	30.234	15.617	31.590	74.264	57.497	5.285	30.237	21.126	30.462	90.007	56.194	10.219
Zma-miR2008	GRMZM2G406101	53.729	36.040	37.020	14.245	8.216	0.262	46.147	37.249	29.654	8.935	6.846	1.009
Zma-miR2016	GRMZM2G471335	0.976	0.776	0.622	0.058	0.000	0.000	2.875	0.976	2.937	0.119	0.117	0.000
Zma-miR2006	GRMZM5G826456	28.752	6.414	23.307	25.515	7.711	2.875	31.825	17.786	12.229	17.560	8.942	4.401
Zma-miR2010	GRMZM5G830319	0.000	0.000	0.045	0.000	0.014	0.078	0.000	0.000	0.000	0.000	0.000	0.024
Zma-miR2009	GRMZM5G862565	12.967	2.486	16.332	6.936	3.416	2.175	16.988	9.428	8.463	5.156	4.960	3.523
Zma-miR2008	GRMZM5G872216	14.396	6.786	13.869	6.343	4.244	1.229	16.516	10.320	13.383	5.423	5.551	2.981
Zma-miR2015	GRMZM5G884280	4.819	1.051	1.912	0.904	0.190	0.045	9.123	2.929	3.652	0.922	0.864	1.098

Figure S1

(A)

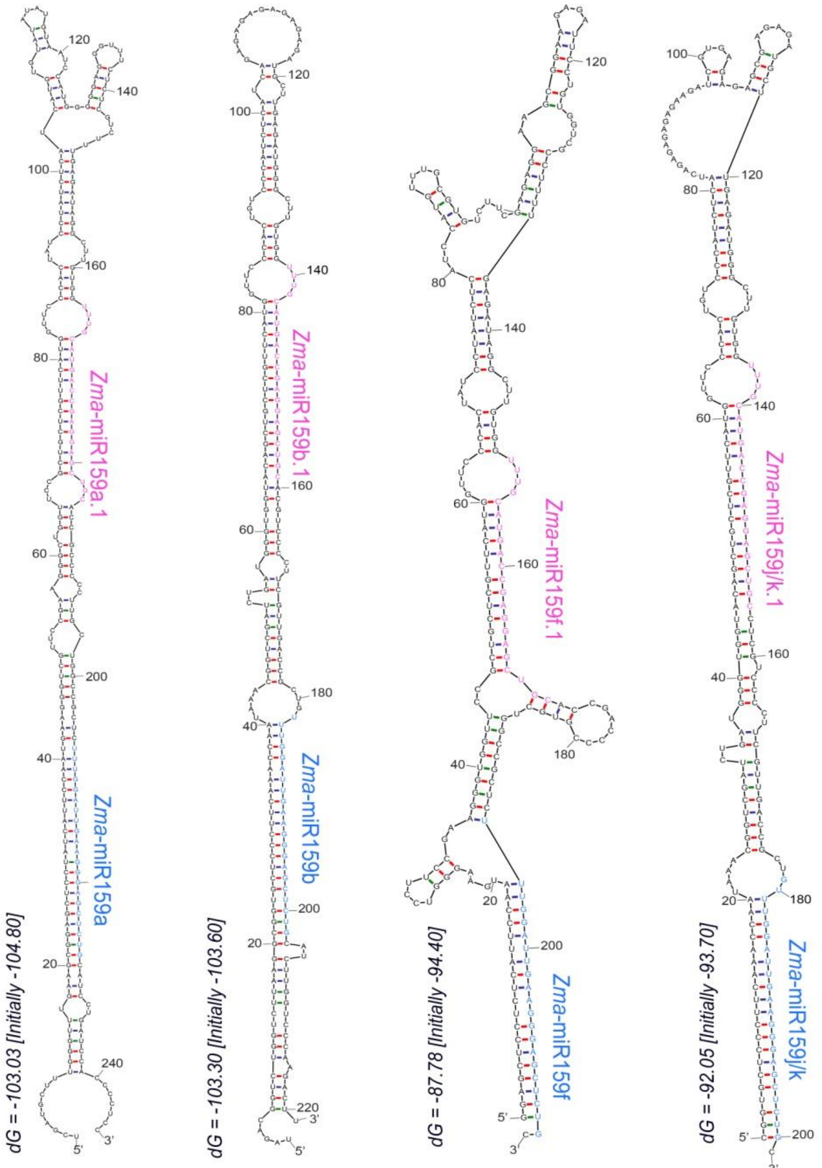

(B)

```
Maize   GAGATAGGCTTGTGGTTTGCATGACCGAGGAGCTGCACCGCCCCCTTGCTGGCCGCTCTTTGGATTGAAGGGAGCTCTGCAT
                                         *
Wheat   GAGAGAGGCCTGTGGTTTGCATGACCGAGGAGCCGCTTCGATCCCTCGCTGACCGCTGTTTGGATTGAAGGGAGCTCTGCAT
                                *        *
Barley  GAGATAGGCTTGTGGTTTGCATGATCGAGGAGCCGCTTCGATCCCTCGCTGACCGCTGTTTGGATTGAAGGGAGCTCTGCAT
                       *        * *
Rice    GGGATAGGCTTATGGCTTGCATGCCCCAGGAGCTGCATCAACCCTACATGGACC.CTCTTTGGATTGAAGGGAGCTCTGCAT
```

Figure S2

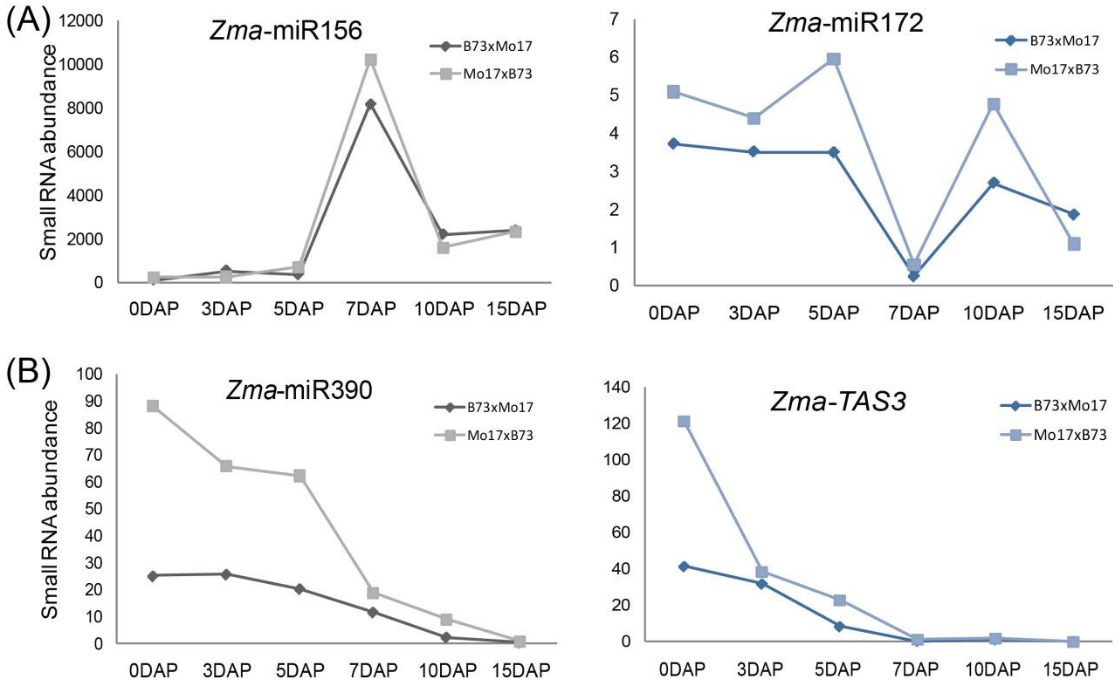
(A)
Zma-miR156
B73xMo17
Mo17xB73
Small RNA abundance
12000
10000
8000
6000
4000
2000
0
0DAP 3DAP 5DAP 7DAP 10DAP 15DAP
Zma-miR172
B73xMo17
Mo17xB73
7
6
5
4
3
2
1
0
0DAP 3DAP 5DAP 7DAP 10DAP 15DAP
(B)
Zma-miR390
B73xMo17
Mo17xB73
Small RNA abundance
100
90
80
70
60
50
40
30
20
10
0
0DAP 3DAP 5DAP 7DAP 10DAP 15DAP
Zma-TAS3
B73xMo17
Mo17xB73
140
120
100
80
60
40
20
0
0DAP 3DAP 5DAP 7DAP 10DAP 15DAP
